# Antiobesity Activities of Methanolic Extracts of* Amaranthus dubius*,* Cucurbita pepo*, and* Vigna unguiculata* in Progesterone-Induced Obese Mice

**DOI:** 10.1155/2017/4317321

**Published:** 2017-08-30

**Authors:** Kathryn Wanjiku Nderitu, Njagi Shadrack Mwenda, Ndegwa John Macharia, Stephen Super Barasa, Mathew Piero Ngugi

**Affiliations:** ^1^Department of Chemistry and Biochemistry, School of Science, University of Eldoret, Eldoret, Kenya; ^2^Department of Biochemistry and Biotechnology, School of Pure and Applied Sciences, Kenyatta University, Nairobi, Kenya

## Abstract

*Amaranthus dubius*,* Vigna unguiculata,* and* Cucurbita pepo* are traditionally used to manage obesity in Kenya but lack scientific validation to support their use. The aim of this study was to determine the antiobesity activity of methanolic leaf extracts of these plants in progesterone-induced obese mice. The activity of the methanolic leaf extracts was orally bioscreened in progesterone-induced obese mice at 200 mg/kg/bw and 400 mg/kg/bw. Body mass index was calculated once per week for four weeks and blood samples were obtained at the end of the experiment for lipid profile analysis. Antiobesity activities of the extracts were compared with the controls. Leaf extracts of* A. dubius*,* C. pepo, *and* V. unguiculata,* at dose concentrations of 200 mg/kgbw and 400 mg/kgbw, showed significant effects on body mass index (*p* < 0.05). There was no significant difference between the three extracts on lipid parameter profiles (*p* > 0.05). The present study showed high food intake in the negative control group as compared with normal control, positive control, and treatment groups. These extracts contained various phytochemicals such as saponins, flavonoids, alkaloids, and steroids and therefore validate use of aforementioned plants in the suppression of obesity and their use for management of obesity is recommended.

## 1. Introduction

Obesity is a serious problem in the world and has been associated with increase in morbidity, mortality, and reduced life expectancy [1]. It occurs as a result of energy imbalance between energy intake and energy expenditure, leading to increased lipid concentration in the blood and enlarged fat mass [2]. Although fat is vital for good health, buildup of a large amount of fat is linked to a variety of health risks such as dyslipidemia, diabetes mellitus, osteoarthritis, hypertension, fatty liver disease, cancers, asthma, and obesity [3, 4].

The prevalence of obesity is increasing rapidly worldwide. Presently, 300 million people are medically obese while more than one billion adults are overweight [5]. WHO also predicted that this number might increase to 3.3 billion by the year 2030. This disease has many factors which contribute to its etiology including sedentary lifestyle such as white collar jobs, lack of physical work out, increase in calorie consumption, endocrine disorders, and psychiatric issues among others [6, 7].

Previous studies also indicate that people increase their intake of high energy snack foods when stressed, thereby leading to obesity [8]. In addition, labor saving devices such as elevators, cars, remote controls, personal computers, and sedentary recreational activities such as watching television, browsing the Internet, and playing video games have highly contributed to obesity in the world [9, 10]. In spite of the urgent need for efficient and safe therapeutics and the probable size of the market for antiobesity drugs, the current efforts for improvement of such drugs are still unsatisfactory [11]. This is due to adverse side effects related to these drugs. More current approaches have focused on natural sources that have been reported to manage obesity and hyperlipidemia as well as reduce weight gain with fewer side effects [12]. Currently, potential use of natural agents for the management of obesity is not fully explored and could be an outstanding substitute approach for developing safe and effective antiobesity drugs. For example, some edible medicinal plants have been used as dietary supplements for body weight management and control in many countries [13, 14]. Such plants include* Camellia sinensis* (L) [15],* Citrus aurantium* L. [16],* Salix matsudana* [17],* Hibiscus sabdariffa* L [18], and* Nelumbo nucifera*, [19] among others.

Most studies have reported that bioactive compounds such as steroids, flavonoids, alkaloids, saponins, and tannins have promising effects in tackling obesity by several mechanisms. Literature survey also indicates that countries that retain African leafy vegetables (ALVs) are not likely to be affected by obesity, diabetes, and cardiovascular diseases. For example, obese mice were introduced to ethanolic extract of* Vigna unguiculata* seeds whose results indicated decreased serum LDL-cholesterol, serum total cholesterol concentration, and serum triacylglycerol level as well as glucose concentration in rats compared with rats fed on high fat control diet. These plants, therefore, showed hypolipidemic and hypoglycemic effects [20]. Another study aimed at evaluating the hypolipidemic and hypoglycemic effects of doses of* Cucurbita pepo* peel extracting male diabetic mice, whose results indicated pumpkin significantly reduced LDL, triglycerides, increased HDL, and reduced glucose levels as compared with the control group [21].* Amaranthus dubius* leaves have also shown antidiabetic effect on alloxan induced diabetic mice [22]. This is due to the bioactive compounds present in them. For example, saponins and flavonoids from* cucurbitae* have been attributed to the hypoglycemic activities in diabetic rats [23]. Alkaloids and flavonoids present in* A. dubius* have also been attributed to its antidiabetic effect [24].

Although these studies provide useful information, no study had been done on* C. pepo*,* V. unguiculata,* and* A. dubius* leaves in relation to their antiobesity effects. This study, therefore, aimed at determining whether methanolic leaf extracts of* A. dubius*,* V. unguiculata,* and* C. pepo* possess antiobesity effects. Progesterone-induced obese mice were used as in vivo models in this study.

## 2. Materials and Methods

### 2.1. Study Site

This study was carried out at the Department of Biochemistry and Biotechnology at the School of Pure and Applied Sciences of Kenyatta University from June, 2016, to September, 2016. Kenyatta University is located 19.3 km from Nairobi city off Thika Super Highway.

### 2.2. Collection and Preparation of the Plant Materials

Fresh leaves of* C. pepo*,* V. unguiculata,* and* A. dubius* were obtained from Marula village, Eldoret subcounty, Uasin Gishu County, Kenya. They were then packaged in khaki bags and transported to Kenyatta University, department of Biotechnology and Biochemistry. Botanical identification and authentication of the plants were done by an acknowledged taxonomist and voucher specimens deposited at the Kenyatta University Herbarium, Nairobi. Voucher specimens numbers assigned were KWO1* (C. pepo)*, KWO2* (V. unguiculata),* and KWO3* (A. dubius)*. The samples were then separately washed using tap water and shade dried for two weeks and later ground into fine homogeneous powder using an electric mill. The powdered plant materials were stored at room temperature away from direct sunlight in closed and dry air tight bags ready for extraction.

### 2.3. Preparation of Methanolic Extracts

Extraction was carried out in the Chemistry departmental Laboratories of Kenyatta University using a protocol described by Grayer and Harborne (1994). Briefly, five hundred grams (500 g) of each powdered plant material was soaked in 1000 ml of methanol at room temperature for 48 hours. Filtration was then done using Whatman number 1 filter paper and later concentrated under vacuum by rotary evaporation at 40°C. The concentrate was then weighed and transferred to an air tight sample bottle and stored at 4°C awaiting use in bioassays.

### 2.4. Experimental Animals

A total of 45 female Swiss albino mice weighing an average of 23 g were used in this study. The animals were housed at the animal handling facility of the Department of Biochemistry and Biotechnology at Kenyatta University. The animals were kept in cages under standard laboratory conditions (25 ± 2°C, 12 h light and 12 h dark cycle). They were then acclimatized to the environmental conditions for one week before the initiation of the experiment. Standard rodent pellets were used to feed the experimental animals and were supplied with water ad libitum. Guidelines by Organization for Cooperation and Development (OECD) and ethics committee of the Kenyatta University on research on animal models were used NACOSTI/P/17/26547/16553.

### 2.5. Induction of Obesity

Obesity was induced in laboratory animals by subcutaneous administration of progesterone (DPMA) at a dose of 10 mg/kgbw. This was done daily, 30 minutes after oral administration of the extracts for 28 days except for the negative controls, which were not administered with the extract [25].

### 2.6. Antiobesity Assay

The mice of female sex were randomly divided into nine groups of five mice each and treated as follows: Group I (normal control) no treatment and Group II (negative control) depo-medroxyprogesterone acetate (DPMA) was administered subcutaneously in the dorsal neck region and the mice received oral administration of water (0.1 ml/mice). Group III (positive control) was given depo-medroxyprogesterone acetate (DPMA) (10 mg/kgbw) subcutaneously at the dorsal neck region and received standard drug orlistat (0.1 ml/mice); Groups IV and V (experimental groups) were given DPMA (10 mg/kgbw) and received* A. dubius *extracts at dosages of 200 mg/kgbw and 400 mg/kgbw, respectively. This design was repeated in* C. pepo* and* V. unguiculata* extracts as summarized in Table 1.

### 2.7. Determination of Body Mass Index

Animal weights and lengths (nasal-anal length) were monitored weekly for 4 weeks using an electronic precision balance and a ruler. To determine body mass index of mice, Lee index was used, which was defined as

(1) $$\begin{array}{c} BMI=\left(\;\frac{\sqrt[{3}]{Bodyweight}}{\text{Nasal-anal length}\;\left(cm\right)}\;\right)\times 1000. \end{array}$$

Mice with BMI ≥ 310 were considered obese [26].

### 2.8. Determination of Food Consumption Pattern

Food consumption behavior of mice was studied on days 7, 14, 21, and 28. The mice were deprived of food 1 hour prior to the experiment. 30 minutes after progesterone administration, 10 g of rat chow was given to groups of mice in their plastic cages and food intake was recorded at 0.5, 1, and 1.5 h intervals [27]. The grams were calculated nearest to 0.1 g with correction to spillage.

### 2.9. Blood Collection and Sera Samples Preparation

At the end of the experimental period (on the 29th day), the tail of each mouse was nipped and venous blood from the tail was collected. Blood glucose test was carried out using a glucose analyzer model (Hypogaurd, Woodbridge, England). The mice were then euthanized using chloroform to minimize stress and pain during sacrificing. The mice were laid on a dissecting board using pins on a bench and then sacrificed. Blood samples were then collected by cardiac puncture and transferred into plain microvacutainer tubes immediately.

The blood samples were then centrifuged at 2400 rpm for 10 minutes to collect clear serum. The clear serum was then aspirated off, packed in Eppendorf tubes and stored frozen at −20°C awaiting analysis. Olympus 640 chemistry auto analyzer was used for analysis of lipid profiles (TG, TC, HDL-C, and LDL-C). All assays were performed based on the standard operating procedures (SOPs) written and maintained at the Department of Biochemistry, Thika Levels Hospital.

### 2.10. Qualitative Phytochemical Screening

The crude extracts were subjected to qualitative phytochemical screening to identify presence or absence of selected bioactive compounds using standard methods [28]. Secondary metabolites tested included alkaloids, terpenoids, diterpenes, flavonoids, phenolics, saponins, anthraquinones, steroids, and tannins.

*Saponins (Froth Test)*. One gram (1 g) of each plant extract was separately added in 2 ml of distilled water in a test tube, sodium bicarbonate solution was added drop wise, and the mixture shaken vigorously. The occurrence of frothing which persisted for at least 15–20 minutes indicated saponins presence.

*Alkaloids*. One gram (1 g) of each plant extract was separately added to 2 ml of 1 molar aqueous concentrated hydrochloric acid. The mixture was stirred and heated in a water bath for 5 minutes and then cooled. Thereafter, the mixture was filtered with Whitman's filter paper number 1 and two drops of Dragendorff's reagent were added. A color change to orange after addition of Dragendorff's reagent indicated presence of alkaloids.

*Terpenoids (Salkowski Test)*. One gram (1 g) of each plant extract was separately added to 1 ml of ethyl acetate/petroleum ether and mixed into 2 ml of chloroform. Three milliliters (3 ml) of concentrated sulphuric acid was added alongside to form a layer. A reddish brown coloration of the interface was formed to show presence of terpenoids.

*Anthraquinones*. Crude extracts were tested for anthraquinones by boiling 1 g of each plant extract with 10% HCl for a few minutes in a water bath. It was then filtered and allowed to cool. Thereafter, 1 ml chloroform (CHCl_3_) and 10% ammonia were added dropwise to the filtrate followed by heating. Rose-pink color formation indicated presence of anthraquinones.

*Flavonoids (Sodium Hydroxide Test)*. Crude extracts were tested for flavonoids by mixing 1 g of each plant extract with 2 ml of diluted sodium hydroxide. An intense golden yellow precipitate indicated presence of flavonoids.

*Steroids*. One gram (1 g) of each plant extract was dissolved in 2 ml of chloroform. Thereafter, 3 ml of concentrated sulphuric acid was added by the sides of the test tube to form a layer. A reddish brown color at the interface indicates the presence of steroids.

*Phenols*. The crude extracts were screened for phenols by adding 1 ml of ferric chloride solution to 1 g of each plant extract. Formation of blue to green color indicated the presence of phenols.

*Tannins*. One milliliter (1 ml) of distilled water was added to each plant extract followed by two drops of 5% iron chloride. Blue-black coloration indicated presence of tannins.

*Diterpenes*. One gram (1 g) of each plant extract was dissolved in water. Thereafter, 3 to 4 drops of copper acetate (Cu(CH_3_COO)_2_) solution were added. Change of color from blue to emerald green indicated presence of diterpenes.

### 2.11. Data Management and Statistical Analysis

Raw data on body mass index and biochemical parameters were tabulated on MS Excel spread sheets where it was organized for statistical analysis. The data was then exported to Minitab statistical software version 17.0 (Minitab Inc., Pennsylvania) for analysis. The data was subjected to descriptive statistics and the results were expressed as mean ± standard error of mean (SEM). One-way analysis of variance (ANOVA) was used to determine whether there were significant differences between the means of different groups. This was followed by Tukey's post hoc tests for pairwise separation and comparison of means. The values of *p* ≤ 0.05 were considered significant. The data was presented in tables and figures.

## 3. Results

### 3.1. Antiobesity Activities of Methanolic Leaf Extracts of* A. dubius*,* C. pepo,* and* V. unguiculata* on Body Mass Index (BMI) in Progesterone-Induced Obese Mice

The methanolic leaf extract of* A. dubius *caused changes in the body mass index (BMI) of progesterone-induced obese mice (Table 2). The body mass index of mice administered with the extract at a dose of 200 mg/kgbw had a similar trend with that of normal control (placebo). The BMI for the mice treated with extract dose of 200 mg/kgbw and normal control were insignificantly different at days 7, 14, 21, and 28 (*p* > 0.05; Table 2). Moreover, mice treated with extract dose of 200 mg/kgbw had a significant decrease in body mass index compared to the mice treated with progesterone (negative control) at days 7, 14, 21, and 28 (*p* < 0.05; Table 2). Mice administered with extract dose of 200 mg/kg/bw had a comparable increase in body mass index with mice treated with orlistat (positive control) at day 7 (*p* > 0.05; Table 2). However, mice treated with 200 mg/kgbw dose of the extract had a significant increase in body mass index compared to mice treated with orlistat (positive control) at days 14, 21, and 28 (*p* < 0.05; Table 2).

At 400 mg/kgbw dose of the extract, the body mass index of mice insignificantly decreased at day 7 compared to the normal controls (*p* > 0.05; Table 2) but there was a significant decrease in body mass index at days 14, 21, and 28 compared to the normal controls (*p* < 0.05; Table 2). There was a significant decrease in body mass index in mice treated with the extract dose of 400 mg/kgbw compared with mice treated with progesterone (negative control) at days 7, 14, 21, and 28 (*p* < 0.05; Table 2). At 400 mg/kgbw dose of the extract, there was no significant difference in BMI compared to the BMI of mice treated with orlistat (positive control) at days 7, 14, 21, and 28 (*p* > 0.05; Table 2).

The methanolic leaf extracts of* C. pepo* also caused changes in the BMI of progesterone-induced obese mice (Table 2). Mice treated with the extract dose of 200 mg/kgbw had an insignificant increase in body mass index at days 7, 14, 21, and 28 compared to normal controls (placebo) (*p* > 0.05; Table 2). However, an insignificant decrease was observed in body mass index of mice administered with extract dose of 200 mg/kgbw compared to mice treated with progesterone (negative control) at days 7, 14, and 21 (*p* > 0.05; Table 2). However, a significant decrease in body mass index was observed at day 28 in mice administered with extract dose of 200 mgkgbw in comparison with mice treated with progesterone (negative control) (*p* < 0.05; Table 2). Mice treated with the dose extract of 200 mg/kgbw significantly increased in body mass index at days 7, 14, 21, and 28 compared to mice treated with orlistat (positive control) (*p* < 0.05; Table 2).

At a dose of 400 mg/kgbw, the body mass index of the mice insignificantly increased in comparison with normal control mice at days 7 and 14 (*p* > 0.05; Table 2). An insignificant decrease in body mass index was observed at day 21 and 28 in mice administered with extract at a dose of 400 mg/kgbw as compared to normal control mice. There was a significant decrease observed in body mass index of mice treated with the extract dose of 400 mg/kgbw at days 7, 21, and 28 compared to the mice treated with progesterone (negative control) (*p* < 0.05; Table 2). However, there was an insignificant decrease in body mass index at day 14 in mice treated with extract dose of 400 mg/kgbw compared to the mice treated with progesterone (negative control) (*p* > 0.05; Table 2). Mice treated with extract dose of 400 mg/kgbw had an insignificant increase in their body mass index at day 7 compared to mice treated with orlistat (positive control) (*p* > 0.05; Table 2). However, the body mass index significantly increased in mice treated with the dose extract of 400 mg/kg bw at days 14, 21, and 28 compared to mice treated with orlistat (positive control) (*p* < 0.05; Table 2).

The methanolic leaf extracts of* V. unguiculata* also caused changes in the BMI of progesterone- induced obese mice. The body mass index of mice administered with the extract dose of 200 mg/kgbw insignificantly increased compared to the normal controls at days 7, 14, 21, and 28 (*p* > 0.05; Table 2). However, the mice treated with extract dose of 200 mg/kgbw had their body mass index insignificantly decrease in comparison with the mice treated with progesterone (negative control) at days 7, 14, 21, and 28 (*p* > 0.05; Table 2). On the other hand, mice treated with extract dose of 200 mg/kgbw showed significant increase in BMI as compared to mice treated with orlistat (positive control) at days 7, 14, 21, and 28 (*p* < 0.05; Table 2).

The body mass index of mice administered with the extract at a dose of 400 mg/kgbw insignificantly increased at day 7 in comparison with normal control mice (*p* > 0.05; Table 2). However, at days 14, 21, and 28, the mice treated with the extract dose of 400 mg/kgbw showed an insignificant decrease in body mass index compared to normal control mice (*p* > 0.05; Table 2). The extract dose of 400 mg/kgbw caused a significant decrease in body mass index at days 7, 14, 21, and 28 compared to mice treated with progesterone (negative control) (*p* < 0.05; Table 2). Mice treated with the extract dose of 400 mg/kgbw resulted in a significant increase in body mass index at day 7 compared to mice treated with orlistat (positive control). Nonetheless, at days 14, 21, and 28, body mass index of mice administered with the extract dose of 400 mg/kgbw had an insignificant increase in body mass index compared to mice treated with orlistat (positive control) (*p* > 0.05; Table 2).

### 3.2. Effects of Methanolic Leaf Extracts of* A. dubius*,* C. pepo,* and* V. unguiculata* on Glucose Levels and Lipid Profiles in Progesterone-Induced Obese Mice

Table 3 shows the effects of methanolic leaf extract of* A. dubius*,* C. pepo,* and* V. unguiculata* on glucose, TG, TC, HDL-C, and LDL-C levels in progesterone-induced obese mice. The mice treated with the extract at a dose of 200 mg/kgbw exhibited a nonsignificant increase in glucose and lipid parameter profiles (TG, TC, HDL-C, and LDL-C) compared to the normal control mice (*p* > 0.05; Table 3). However, mice treated with the extract at a dose of 200 mg/kgbw depicted an insignificant decrease in glucose and lipid parameter profile (TG, TC and LDL-C) levels compared to the mice treated with progesterone (negative control) (*p* > 0.05; Table 3). On the other hand, mice treated with the extract at a dose of 200 mg/kgbw had a nonsignificant increase in glucose, TG, and LDL-C levels compared to mice treated with orlistat (positive control) (*p* > 0.05; Table 3). Mice treated with the extract at a dose of 200 mg/kgbw and orlistat had comparable TC and HDL-C parameters profiles (*p* > 0.05; Table 3).

At the extract dose of 400 mg/kgbw, an insignificant increase in glucose and lipid parameter profiles (TG, TC, HDL-C, and LDL-C) was observed compared to normal mice (*p* > 0.05; Table 3). A nonsignificant decrease in TG, TC, HDL-C, and LDL-C was observed in mice treated with extract dose of 400 mg/kgbw when compared to mice administered with progesterone (negative control). Comparably, there were no significant differences in TG, TC, and HDL-C profiles between mice treated with 400 mg/kgbw of the extract and those treated with orlistat (positive control) (*p* > 0.05; Table 3).

For* C. pepo*, the mice treated with the extract dose of 200 mg/kg/bw showed a nonsignificant increase of glucose, TG, HDL-C, and LDL-C compared to the normal control mice (*p* > 0.05; Table 3). There was an insignificant decrease in glucose, TC, and LDL in mice treated with extract at a dose of 200 mg/kgbw compared to the mice treated with progesterone (negative control) (*p* > 0.05; Table 3). In addition, mice treated with extract dose of 200 mg/kgbw showed a slight increase in glucose and TG compared to mice treated with orlistat (positive control) (Table 3). However, a slight insignificant decrease in TC and LDL-C was observed in mice treated with the extract dose of 200 mg/kgbw compared to mice treated with orlistat (positive control) (*p* > 0.05; Table 3). Similarly, the mice treated with the extract at a dose of 400 mg/kgbw showed similar results to those administered with the extract dose of 200 mg/kgbw as compared to the controls (Table 3).

For* V. unguiculata*, the mice administered with the extract at dose of 200 mg/kg/bw showed an insignificant increase in glucose, TG, TC, HDL-C, and LDL-C levels compared to the normal control mice (*p* > 0.05; Table 3). There was a nonsignificant decrease observed in mice treated at the extract dose of 200 mg/kgbw in glucose, TG, and TC parameters in relation to mice treated with progesterone (negative control). However, an insignificant increase in LDL-C was observed in mice treated at dose level of 200 mg/kgbw compared to mice treated with progesterone (negative control) and orlistat (positive control) (*p* > 0.05; Table 3). At extract dose of 200 mg/kgbw, an insignificant increase in HDL-C levels was observed in mice in comparison with the negative control mice. Similarly, mice treated with orlistat (positive control) showed an insignificant increase in HDL-C as compared to mice treated with progesterone (negative control) (*p* > 0.05; Table 3).

At the extract dose of 400 mg/kgbw, a nonsignificant increase in glucose, TG, TC, HDL-C, and LDL-C levels was observed compared to normal control mice (*p* > 0.05; Table 3). Mice treated with extract dose of 400 mg/kgbw had an insignificant decrease in their glucose, TC, TG, and LDL-C levels compared to mice treated with progesterone (negative control) (*p* > 0.05; Table 3). However, there was an insignificant increase in HDL-C levels in mice administered with the extract dose of 400 mg/kgbw compared to mice treated with progesterone (negative control). Nonsignificant decrease in TC, TG, and LDL-C levels was observed in mice treated with extract dose of 400 mg/kgbw and an insignificant increase in HDL-C when compared with mice treated with orlistat (positive control) (*p* > 0.05; Table 3).

### 3.3. Effects of Methanolic Leaf Extracts of* A. dubius*,* C. pepo,* and* V. unguiculata* on Food Consumption Pattern in Progesterone-Induced Obese Mice

**Figure 1:**
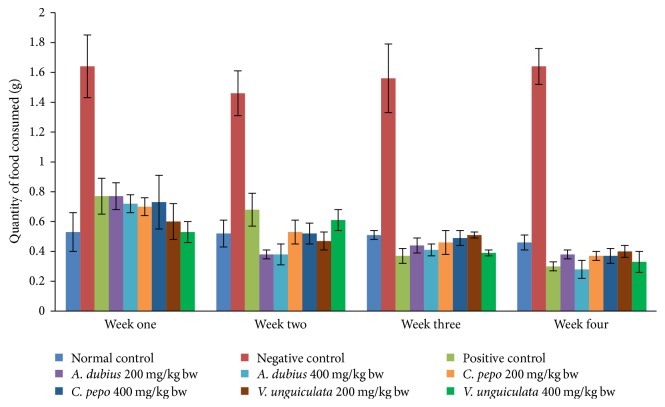
Effect of methanolic leaf extract of* A. dubius*,* C. pepo,* and* V. unguiculata* on food consumption pattern in progesterone-induced obese mice.

Generally the results show that the progesterone-induced obese mice (negative controls) had high food intake in comparison with the mice in normal control group, mice treated with the plant extracts (200 mg/kgbw and 400 mg/kgbw), and orlistat treated mice positive control (Figure 1).

### 3.4. Qualitative Phytochemical Composition of Methanolic Extracts of* A. dubius, C. pepo,* and* V. unguiculata*

Qualitative phytochemical screening of the Methanolic leaf extracts of* V. unguiculata*,* C. pepo, *and* A. dubius* showed presence of alkaloids, steroids, saponins, phenolics, flavonoids, terpenoids, and diterpenes. However, tannins and anthraquinones were absent (Table 4).

## 4. Discussion

Obesity is defined as a chronic metabolic disorder that is characterized by increased lipid concentration and enlarged fat mass. It is a result of imbalance between energy expended and energy taken in [24]. At the cellular level, it is characterized by an increase in size and number of adipocytes differentiated from fibroblastic preadipocytes in adipose tissues. Furthermore, obesity has led to reduced life expectancy and health problems such as obstructive sleep apnea, type 2 diabetes, and cardiovascular diseases among others [29].

Many attempts have been made to correct obesity by designing a number of drugs such as orlistat, fibrates, and sibutramine. However, they have been found to have severe side effects and are unaffordable [30]. Thus, the importance for development of herbal formulations has been overemphasized [24]. For example, a variety of natural products such as isolated plant compounds and crude plant extracts have been reported to cause body weight reduction. Therefore, they have been largely used in managing obesity [31].

This study was carried out to evaluate the antiobesity activities of* A. dubius*,* C. pepo,* and* V. unguiculata* in progesterone-induced obese female mice. Progesterone stimulates hyperphagia through the progestin receptors, which have been reported to be expressed on the serotonergic neurons [32]. The conventional antiobesity drug (orlistat), other than being a pancreatic lipase inhibitor, suppresses hyperphagia by altering the main central nervous system appetite monoamine neurotransmitters by slowing down the reuptake of 5-HT (serotonin) at the hypothalamic site, which regulates food consumption [33]. It also reduces ghrelin hormone which increases appetite [34]. This suggests a possible interaction between serotonin receptor system and the neurosteroid in regulating body weight and food ingestion.

Furthermore, studies report that the ovarian hormone level disturbances may predispose females to binge eating by causing alterations in the serotonergic receptor function or serotonin level [27]. Findings of this study agree with other studies, which reported progesterone as the most fattening of steroid hormones and also agree with a study that reported weight gain in users of depo-medroxyprogesterone acetate (DMPA) [35, 36]. This was evident in mice treated with progesterone (negative control), which showed the highest BMI throughout the study period compared to normal controls and other treatment groups of mice.

The mice given* A. dubius*, at a dose of 400 mg/kg body weight, significantly decreased in BMI from the first to the last week of the experiment. This was in contrast with mice given extract dose of 200 mg/kg body weight of* A. dubius, *which showed an increase in BMI. Thus, this study established that* A. dubius *at a dose extract of 400 mg/kg body weight had beneficial effect in management of obesity as opposed to the extract dose of 200 mg/kg body weight. Comparably, work carried out by [27] demonstrated antiobesity effects of* Stellaria *media against progesterone-induced obesity in Swiss albino mice when administered at a dose of 400 mgkg/bodyweight.

Similarly,* C. pepo* extracts caused a dose dependent response against BMI in mice. At a dose of 200 mg/kg body weight,* C. pepo* extract did not show any decrease in BMI throughout the study period. However, at a dose level of 400 mg/kg body weight, BMI decreased sequentially starting from the third week. Therefore, in this study* C. pepo* extract at a dose level of 400 mg/kgbw was established to have antiobesity effects. On the other hand, mice treated with* V. unguiculata* extracts at dose of 200 mg/kg body weight and 400 mg/kg body weight indicated a sequential decrease in BMI from the second week to the last week of the study period. Therefore, this study established that* V. unguiculata* extract at dose levels of 200 mg/kgbw and 400 mg/kgbw had beneficial effect in the management of obesity.

The antiobesity activities of the methanolic leaf extract of* A. dubius*,* C. pepo,* and* V. unguiculata* might be due to the action of phytoconstituents present in them. In fact, the antiobesity activity may be attributed to the synergy of a variety of bioactive compounds in the extracts [37]. In the present study, phytochemical screening indicated presence of alkaloids, flavonoids, steroids, diterpenes, saponins, and phenols.

Many studies have reported plants with phytochemicals like flavonoids, alkaloids, saponins, tannins, steroids, and phenols to have antiobesity effects. Flavonoids activate *β*-adrenergic receptors which are involved in the burning of fats by exhibiting PPAR-*γ* ligand binding activity, similar to PPAR-*γ* agonists [38]. Other studies also suggest that flavonoids inhibit adipogenesis and induce apoptosis of 3T3-L1 preadipocytes in mice [39].

Similarly, flavonoids and phenols have been reported to function as antioxidants, thus preventing diseases such as obesity by modulating oxidative stresses in the body [40]. Free radicals such as nitrogen and oxygen species, which are cell metabolism byproducts in humans, can lead to diverse life-threatening ailments such as obesity, coronary heart diseases, and Type II diabetes mellitus [41].

In many other studies, alkaloids present in plant extracts significantly reduce the expression levels of several adipocyte marker genes including enhancer binding proteins and proliferator activated receptor hence inhibiting adipogenesis [42]. Thus, in the current study, such attestations might have led to the antiobesity activities of* A. dubius*,* C. pepo,* and* V. unguiculata*.

There is considerable proof that obese persons are at a higher risk of developing many diseases such as hypertension, hyperlipidemia, cardiovascular diseases, and type 2 diabetes mellitus [43, 44]. Alterations of carbohydrates, proteins, and lipid metabolism are also significant factors in the development of diabetes mellitus and cardiovascular diseases. Previous studies show that increase in serum triglyceride and cholesterol levels may be risk factors for development of cardiovascular diseases [45]. For this reason, several analyses were done in this study, which included glucose and lipid profile analysis in obese mice.

Although the mice were found to be obese, this study showed an insignificant difference in glucose levels and lipid parameter profiles among all the treated mice in comparison with the negative controls. In addition, an increase in triglyceride was observed in the obese mice while a slight decrease was observed with supplementation of the extracts. Therefore, the study agrees with the findings of [40], who did a study on antiobesity activity of* Moringa oleifera* in obese mice and found out that its crude extract possesses hypotriglycemic activity. Consequently, it is likely that the phytochemicals identified as occurring in the methanolic extracts of* A. dubius*,* V. unguiculata,* and* C. pepo* are responsible for their hypotriglycemic activity. Few studies reported that saponins and flavonoids result in reduction of triglycerides and total cholesterol by formation of large micelles excreted in bile. These bioactive compounds are also said to decrease absorption of cholesterol in the intestines and serum levels of low density lipoprotein cholesterol [46]. Thus, this study postulates that presence of saponins and flavonoids in the methanolic extracts of the three ALVs could result in the reduction in triglyceride and cholesterol.

The presence of sterols in the three ALVs could also result in cholesterol and low density lipoprotein reduction in serum. Studies have suggested that phytosterols inhibit cholesterol absorption by competing with cholesterol for micelle formation in the intestinal lumen [47]. Esterification of cholesterol occurs in the enterocytes in a reaction catalyzed by acetyl-coA acetyltransferase-2 enzyme. Esterified cholesterol is then packed into chylomicrons and later transferred into the lymphatic system. Nonesterified cholesterol and phytosterols are pumped back into the intestinal lumen by the ABC transporters. This reduces the sum of cholesterol assimilated in the system [48]. Clinical studies have also reported that 15% of phytosterols lower LDL-cholesterol [49].

Other studies have also reported that presence of phytosterols reduces the serum levels of TG by inhibiting accumulation of TG and expression of the lipogenic genes [50]. Phytosterols also increase high density lipoproteins [51], while flavonoids are reported to inhibit triglycerides accumulation by inhibiting pancreatic lipase activity [52, 53].

A variety of studies have been done with regard to progesterone and its hyperphagic effect. A study conducted by [35] reported that subchronic treatment with progesterone for four weeks significantly increased food intake in female mice. In this study, this was evident in negative control mice, which showed a higher increase rate of food consumption as compared to normal controls. After 30 minutes, 1 hour, and 1.5 hours of subchronic treatment with progesterone, food consumption was significantly decreased by the coadministration of methanolic leaf extracts of* A. dubius*,* C. pepo,* and* V. unguiculata* at dose levels of 200 mg/kgbw and 400 mg/kgbw.

Phytoconstituents such as flavonoids and saponins have been reported to increase weight loss by increasing energy expenditure and reducing food intake. This occurs by induction of anorectic effect in the hypothalamus, through stimulation of the capsaicin-sensitive sensory nerves, possibly vagal afferent nerves leading to reduced body weight gain and food consumption [54, 55]. Thus, this study postulates that saponins and flavonoids present in the African leafy vegetables methanolic extracts resulted in the reduction of food intake in experimental groups.

In addition, presence of alkaloids has been shown in previous studies to reduce food intake, increase food expenditure, and suppress appetite by increasing norepinephrine hormone [56]. Similarly, previous studies have reported sterols to have anorexiant properties that enhance satiation and reduce absorption of fats by increasing serum serotonin levels [57]. Thus, this study postulates that the presence of those phytochemicals led to the reduction of food consumption.

From this study, it is also postulated that saponins and flavonoids absorption from gastrointestinal tract increased the leptin hormone sensitivity, crossed the blood brain barrier (BBB), and entered in the brain and, upon reaching the brain, acted on receptors in the hypothalamus to curtail appetite [58]. It is also apparent that saponins and flavonoids inhibited neuropeptide Y (NP-Y) in the hypothalamus [54].

In addition, this study postulates that hypothalamic neural networks downstream from the leptin receptor were affected, such as the *α*-melanocyte-stimulating hormone (*α*-MSH) pathway, leading to a reduction of the arcuate hypothalamic *α*-MSH immunoreactivity and leptin hormone. Therefore, the decreased leptin level probably associated with methanolic leaf extracts of African leafy vegetable supplementation may be attributed to the decrease of adipose tissue and thus the antiobesity effect [59]. Thus, this study hypothesizes decrease in adipose tissue as a result of the inhibitory effect of methanolic extracts of ALVs on the differentiation of 3T3-L1 preadipocytes into adipocytes through downregulation expression of PPAR*γ* [60].

## 5. Conclusion

We conclusively demonstrate that methanolic leaf extracts of* A. dubius*,* C. pepo,* and* V. unguiculata* have antiobesity activity and reveal the presence of vital phytochemicals. The antiobesity activity of the studied plant may have resulted from its phytochemicals constituents. It provided evidence that these extracts decrease body mass index, food intake, and lipid levels in serum in progesterone-induced obese mice. The study, therefore, confirmed the use of African leafy vegetables in prevention and management of obesity. Consideration should be made to subject the plant to a more nonpolar solvent extraction and compare activities of both methanolic and nonpolar solvents. A bioassay guided fractionation should also be done.

## Figures and Tables

**Table 1:** Experimental design.

Treatment groups	Treatment	Number of mice
I (Normal group)	Water ad libitum	5
II (Negative control)	Depo-medroxyprogesterone acetate (DMPA) (10 mg/kgbw)	5
III (Positive control)	DMPA (10 mgkgbw) + (orlistat) (10 mg/kgbw)	5
IV	DMPA (10 mg/kgbw) + extracts (200 mg/kgbw)	5
V	DMPA (10 mg/kgbw) + extracts (400 mg/kgbw)	5

**Table 2:** Effects of methanolic leaf extract of *A. dubius*, *C. pepo,* and *V. unguiculata* on body mass index in progesterone-induced obese mice.

Treatment	Time (Days)				
	0	7	14	21	28
Normal control	297.37 ± 2.01^a^	301.38 ± 1.95^b^	302.31 ± 1.64^b^	302.72 ± 1.10^b^	303.91 ± 0.76^b^
Progesterone (10 mg/kg bw)	303.41 ± 1.48^a^	312.48 ± 2.34^a^	313.97 ± 1.94^a^	316.41 ± 1.84^a^	317.49 ± 1.79^a^
Orlistat (10 mg/kg bw)	301.16 ± 1.02^a^	295.70 ± 2.06^b^	292.49 ± 1.91^c^	290.48 ± 1.69^c^	288.40 ± 1.97^c^
*A. dubius* (200 mg/kgbw)	299.84 ± 0.91^a^	302.69 ± 0.56^b^	305.53 ± 0.99^b^	305.53 ± 2.66^b^	307.89 ± 1.99^b^
*A. dubius* (400 mg/kgbw)	301.84 ± 1.13^a^	296.34 ± 2.93^b^	291.60 ± 2.49^c^	291.90 ± 3.35^c^	290.11 ± 2.44^c^
*C. pepo* (200 mg/kgbw)	300.01 ± 0.43^a^	308.11 ± 1.55^ab^	308.56 ± 1.62^ab^	308.23 ± 2.58^ab^	308.39 ± 1.10^b^
*C. pepo* (400 mg/kgbw)	301.08 ± 1.32^a^	303.21 ± 2.28^bc^	305.47 ± 3.52^ab^	300.95 ± 2.80^b^	300.55 ± 3.66^b^
*V. unguiculata* (200 mg/kgbw)	299.70 ± 0.96^a^	307.46 ± 1.01^ab^	306.79 ± 0.98^ab^	304.96 ± 3.05^ab^	304.92 ± 3.21^ab^
*V. unguiculata* (400 mg/kgbw)	300.65 ± 0.77^a^	306.90 ± 1.90^ab^	299.18 ± 5.98^bc^	299.63 ± 6.01^bc^	296.59 ± 8.58^b^

Values expressed as Mean ± SEM for five animals per group. Statistical comparisons were made within a column and values with the same superscript letter are not significantly different by one-way ANOVA followed by Tukey's post hoc test (*p* > 0.05).

**Table 3:** Effects of methanolic leaf extracts of the plants on glucose levels and lipid profiles in progesterone-induced obese mice.

Treatment	Biochemical Parameters				
	Glucose	TG	TC	HDL-C	LDL-C
Normal	5.12 ± 0.27^a^	0.74 ± 0.11^a^	1.08 ± 0.16^a^	0.52 ± 0.19^a^	0.22 ± 0.03^a^
Progesterone (10 mg/kgbw)	6.58 ± 0.28^a^	1.00 ± 0.2^a^	1.78 ± 0.36^a^	0.54 ± 0.22^a^	0.40 ± 0.08^a^
Orlistat (10 mg/kgbw)	5.54 ± 0.30^a^	0.92 ± 0.08^a^	1.50 ± 0.15^a^	0.60 ± 0.07^a^	0.42 ± 0.15^a^
*A. dubius* (200 mg/kg/bw)	6.32 ± 0.76^a^	0.96 ± 0.07^a^	1.50 ± 0.09^a^	0.60 ± 0.07^a^	0.54 ± 0.14^a^
*A. dubius* (400 mg/kg/bw)	5.20 ± 0.22^a^	0.72 ± 0.10^a^	1.78 ± 0.22^a^	0.74 ± 0.24^a^	0.40 ± 0.04^a^
*C. pepo* (200 mg/kg/bw)	5.80 ± 0.19^a^	1.00 ± 0.06^a^	1.08 ± 0.08^a^	0.58 ± 0.16^a^	0.22 ± 0.03^a^
*C. pepo* (400 mg/kg/bw)	5.78 ± 0.97^a^	0.94 ± 0.04^a^	1.24 ± 0.16^a^	0.54 ± 0.09^a^	0.28 ± 0.02^a^
*V. unguiculata* (200 mg/kg/bw)	5.52 ± 0.56^a^	0.88 ± 0.05^a^	1.36 ± 0.04^a^	0.60 ± 0.00^a^	0.62 ± 0.15^a^
*V. unguiculata* (400 mg/kg/bw)	5.32 ± 0.17^a^	0.82 ± 0.06^a^	1.28 ± 1.02^a^	0.62 ± 0.03^a^	0.26 ± 0.05^a^

Values expressed as Mean ± SEM for five animals per group. Statistical comparisons were made within a column and values with the same superscript letter are not significantly different by one-way ANOVA followed by Tukey's post hoc test (*p* > 0.05).

**Table 4:** Qualitative phytochemical screening of methanolic leaf extract of* V. unguiculata*, *C. pepo,* and *A. dubius*.

Phytochemicals	*V. unguiculata*	*C. pepo*	*A. dubious*
Flavonoids	+	+	+
Steroids	+	+	+
Alkaloids	+	+	+
Tannins	−	−	−
Anthraquinones	−	−	−
Saponins	+	+	+
Terpenoids	+	+	+
Diterpenes	+	+	+
Phenols	+	+	+

Present phytochemicals are denoted by (+) sign; absent phytochemicals are denoted by (−) sign.
